# *EIF2AK4* mutation as “second hit” in hereditary pulmonary arterial hypertension

**DOI:** 10.1186/s12931-016-0457-x

**Published:** 2016-11-04

**Authors:** Christina A. Eichstaedt, Jie Song, Nicola Benjamin, Satenik Harutyunova, Christine Fischer, Ekkehard Grünig, Katrin Hinderhofer

**Affiliations:** 1 Center for Pulmonary Hypertension at the Thoraxclinic, University Hospital Heidelberg, Heidelberg, 69126 Germany; 2 Institute of Human Genetics, Heidelberg University, Im Neuenheimer Feld 366, 69120 Heidelberg, Germany; 3 Translational Lung Research Center Heidelberg (TLRC), German Center for Lung Research (DZL), Heidelberg, Germany

**Keywords:** Hereditary pulmonary arterial hypertension, Next generation sequencing, Pulmonary veno-occlusive disease, Two-gene model

## Abstract

**Background:**

Mutations in the *eukaryotic translation initiation factor 2α kinase 4* (*EIF2AK4*) gene have recently been identified in recessively inherited veno-occlusive disease. In this study we assessed if *EIF2AK4* mutations occur also in a family with autosomal dominantly inherited pulmonary arterial hypertension (HPAH) and incomplete penetrance of *bone morphogenic protein receptor 2* (*BMPR2*) mutations.

**Methods:**

Clinical examinations in a family with 10 members included physical examination, electrocardiogram, (stress)-echocardiography and lung function. Manifest PAH was confirmed by right heart catheterisation in three affected subjects. Genetic analysis was performed using a new PAH-specific gene panel analysis with next generation sequencing of all known PAH and further candidate genes. Identified variants were confirmed by Sanger sequencing.

**Results:**

All living family members with manifest HPAH carried two pathogenic heterozygous mutations: a frame shift mutation in the *BMPR2* gene and a novel splice site mutation in the *EIF2AK4* gene. Two family members who carried the *BMPR2* mutation only did not develop manifest HPAH.

**Conclusions:**

This is the first study suggesting that *EIF2AK4* can also contribute to autosomal dominantly inherited HPAH. Up to now it has only been identified in a recessive form of HPAH. Only those family members with a co-occurrence of two mutations developed manifest HPAH. Thus, the *EIF2AK4* and *BMRPR2* mutations support the “second hit” hypothesis explaining the variable penetrance of HPAH in this family. Hence, the assessment of all known PAH genes in families with a known mutation might assist in predictions about the clinical manifestation in so far non-affected mutation carriers.

## Background

Pulmonary arterial hypertension (PAH) is a rare disease characterised by an elevated pulmonary artery mean pressure caused by the obstruction of small pulmonary arteries leading to right heart failure. The disease may occur sporadically (idiopathic), associated with other diseases or in a heritable form. Hereditary pulmonary arterial hypertension (HPAH) is considered an autosomal dominantly inherited disease with incomplete penetrance. Mutations in the *bone morphogenic protein receptor type 2* (*BMPR2*) gene have been established as the most common cause of PAH since the discovery of the first mutations in the year 2000 [1–5]. Up to 87 % of HPAH families and 25 % of idiopathic PAH patients show a genetic defect in *BMPR2* [6, 7]. Further mutations of genes within the *BMPR2* pathway may lead to PAH manifestation [8] for example in the co-receptors *ACVRL1* and *ENG*. Through the advent of new sequencing technologies, such as the next generation sequencing (NGS) the sequencing of entire exomes became feasible. This led to the discovery of new PAH causing genes outside the canonical BMPR2 pathway such as the potassium channel *KCNK3* [9] or cell membrane protein *CAV1* gene [10].

In the same manner, mutations in the *eukaryotic translation initiation factor 2α kinase 4* (*EIF2AK4*) gene have recently been identified as disease causing in families with recessively inherited veno-occlusive disease [11] and pulmonary capillary haemangiomatosis [12]. Both diseases are classified as a subgroup of pulmonary hypertension next to PAH in the current guidelines [13]. Up to date only one specific mutation within *EIF2AK4* has been identified in 6 families with autosomal recessive PAH from the same itinerant Iberian community [14, 15]. The penetrance of *BMPR2* mutations is age dependent and has been estimated to range between 27 % in women and 14 % in men at the time of first PAH diagnosis [16] but may be as high as 43–50 % in some families [17, 18]. Thus, not all mutation carriers will develop the disease during their life time. The causes for this phenomenon still remain unclear. Possible modifiers which lead to disease manifestation could be additional mutations in the same pathway [19–21], so called “second hits” or other external stimuli.

In this study, we therefore investigated the penetrance in a HPAH family with a known *BMPR2* mutation by a careful clinical and genetic assessment. Our aim was to elucidate whether we can detect further autosomal dominantly inherited gene defects in this family which may explain the clinical disease manifestation and incomplete penetrance.

## Methods

### Subjects and clinical characterisation

Members of a family with autosomal dominantly inherited HPAH were clinically and genetically assessed. All living genetically related family members were invited to participate. After written informed consent was obtained family members underwent clinical assessment and genetic counselling. A three generation pedigree was drawn including nine family members of the index patient. EDTA-blood was taken for genetic analysis.

Clinical procedures consisted of recording the family and medical history, physical examination, laboratory parameters including N-type pro brain natriuretic peptide (NT-proBNP), 12-lead electrocardiogram, lung function test, arterial blood gases, 6-min walking distance, echocardiography, stress-Dopplerechocardiography and cardiopulmonary exercise testing as described previously [22]. High resolution computer tomography of the lung was conducted to exclude pulmonary veno-occlusive disease. Left heart catheterisation was performed in all patients with suspected left heart diseases and when clinically indicated. Manifest HPAH was diagnosed according to the current guidelines [13]. Right heart catheterisation was performed in the living HPAH patients to confirm diagnosis and for follow-up.

### Genetic assessment

Genomic DNA was isolated from peripheral blood using a salting out procedure [23] (Autopure, LGC, Germany). Sanger sequencing for *BMPR2* (ENST00000374580) was conducted in the index patient using Big Dye Terminator V1.1 cycle sequencing kit and ABI 3130xl genetic analyzer (ThermoFisher Scientific, USA). Duplications and deletions were screened by multiplex ligation-dependent probe amplification (MLPA, kit P093-C2, MRC-Holland, the Netherlands).

A new PAH gene panel diagnostic based on next generation sequencing was designed to analyse second hit mutations in 11 PAH (*ACVRL1* or *ALK1*, *BMPR1B*, *BMPR2*, *CAV1*, *EIF2AK4*, *ENG*, *KCNA5*, *KCNK3*, *SMAD1*, *SMAD4*, *SMAD9*) and 20 further candidate genes in the index patient. DNA was enriched with a customised SureDesign panel (Agilent Technologies, USA) and sequenced on the MiSeq (Illumina, USA). Exonic regions and exon-intron boundaries were analysed with SeqPilot 4.1.2 (JSI medical systems GmbH, Germany). Variants were characterised following the recommendations of the Human Genome Variation Society (HGVS version 2.15.11) [24]. Non-synonymous missense variants with a population frequency <5 % were assessed regarding their evolutionary conservation, location within functional gene domains and functional consequence using four *in silico* prediction programs: MutationTaster, SIFT, Align GVGD and PolyPhen2 implemented in Alamut Visual 2.7.1 (interactive biosoftware, France). Variants were confirmed by Sanger sequencing in the index patient and assessed in family members to clarify mutation status. Any variants disrupting gene function were considered mutations.

### Functional assessment of mutations

RNA was isolated from EDTA blood using standard procedures. Copy DNA (cDNA) was generated with a reverse transcriptase reaction adding hexamers for 10 min at 65 °C, a cDNA-Mix (Invitrogen, USA) for 2 h at 37 °C and a final reaction for 10 min at 65 °C.

**Fig. 1:**
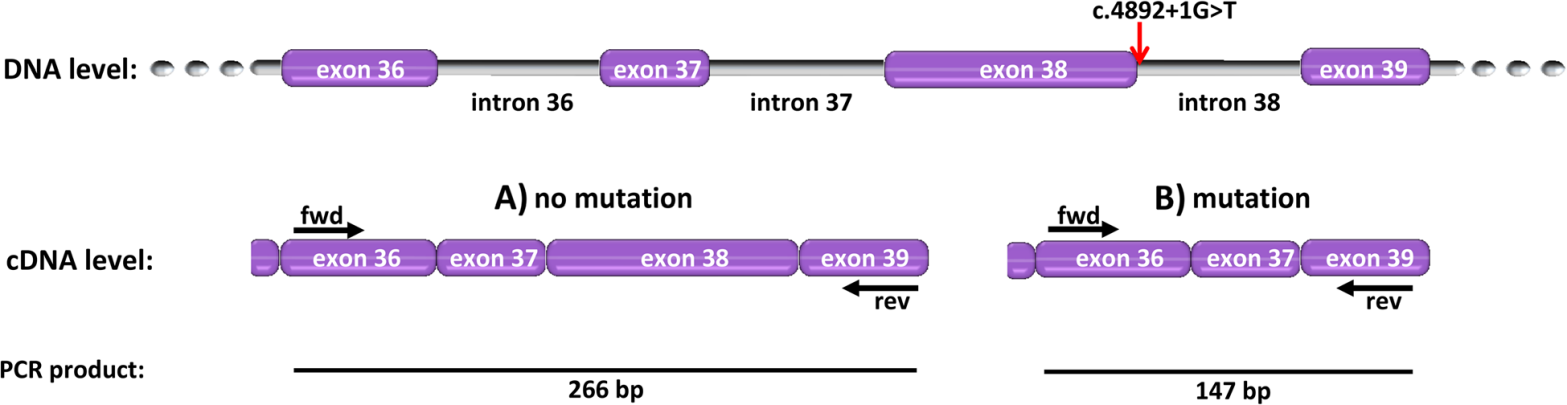
Functional assessment of splice site mutation. The top level displays the location of the exons 36-39 of the *EIF2AK4* gene within the genome including intronic sequences. The mutation (c.4892+1G>T) is indicated by the *red arrow*. The second level displays copy DNA (cDNA) generated by a reverse transcriptase from extracted messenger RNA. *A)* shows the intact sequence and PCR product if no mutation is present, including exon 38. *B)* displays the effect of the mutation leading to a loss of exon 38. Exon sizes are proporational to their respective number of amino acids

A PCR was designed to assess the cDNA corresponding to the messenger RNA of *EIF2AK4* (ENST00000263791, NM_001013703)*.* The PCR product span the exon 38 using a forward primer annealing in exon 36 (5′ GACCTCCCTTGCCAACTTAC 3′) and a reverse primer annealing in exon 39 (5′ AGAT TCTGTAGTAGTCATCTCTATAGC 3′). The expected size of the intact PCR product was 266 bp and the one of the PCR product skipping exon 38 was 147 bp. Primer annealing and effect of the splice site mutation are displayed in Fig. 1. cDNA was denatured for 5 min at 95 °C, and subsequently amplified in 35 cycles (1 min at 95 °C, 1 min at 56.5 °C, 1.5 min at 72 °C; final elongation 10 min at 72 °C). While several transcripts exist for *EIF2AK4* the considered isoform is the most common one in humans since it is referred to as reference sequence by NCBI RefSeq. NCBI RefSeq is a database which we used as reference standard for reporting the location of medically important variations. PCR products were sequenced with Sanger sequencing to identify the altered base pair sequence of the messenger RNA.

### Statistical analyses

Significance of co-occurance of *BMPR2* and *EIF2AK4* was calculated by using an estimated heterozygote frequency of *EIFAK4* mutations retrieved from the Exome Aggregation Consortium (ExAC) database [25] and taking into account the number of genes on the panel by correcting for multiplicity. A *p*-value lower than 5 % was considered statistically significant.

## Results

### Clinical characterisation

**Fig. 2:**
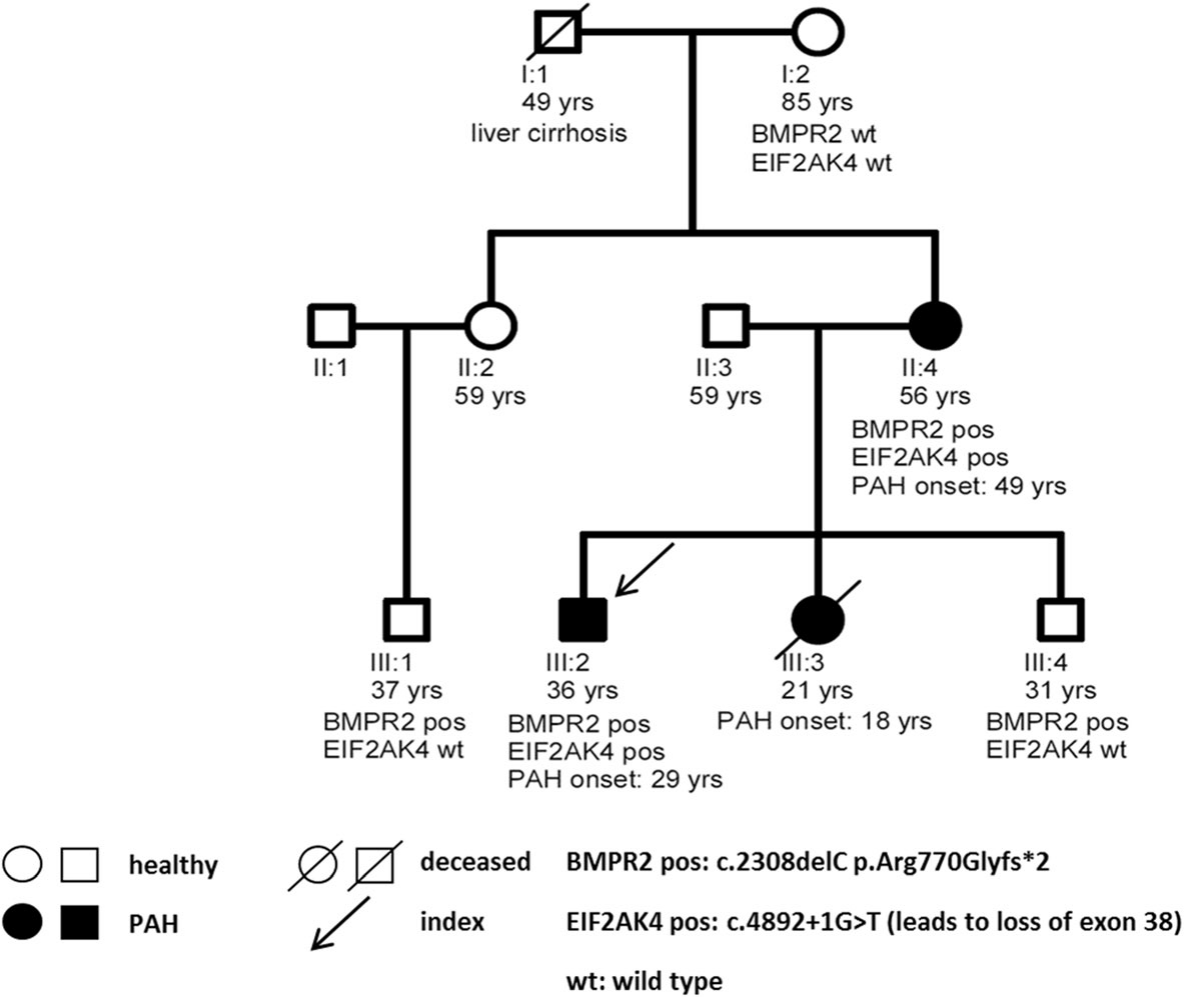
Pedigree of HPAH family. Affected individuals have *filled symbols*, healthy individuals *empty symbols*. Age, mutation status and age of PAH onset are provided below. Patient III:3 received a lung transplantation one year after diagnosis and died of transplant rejection one year later. The *BMPR2* mutation leads to a premature stop codon and the *EIF2AK4* mutation to a splice site change and subsequent loss of exon 38 presumably followed by a frame shift and premature insertion of a stop codon

Clinical and genetic data are presented in Fig. 2, clinical parameters in Table 1. PAH was excluded in healthy family members by regular clinical assessments including physical examination, lung function tests, electrocardiogram, and in particular by echocardiography, stress-Doppler-echocardiography, spiroergometry and NT-proBNP values. Diagnosis of PAH was confirmed by right heart catheterisation in patients II:4, III:2 and III:3. The disease was very severe in all three patients but could be stabilised with medication in patients II:4, and III:2. Patient III:3 showed a rapid progression and received a lung transplantation 1.5 years after diagnosis. She died only one year after transplantation due to a rejection of the donor organ (Fig. 2). Patient II:4 and III:2 were under dual therapy at the time of this writing. Stable haemodynamic parameters were observed in the latest catheter of patient II:4 with a mean pulmonary artery pressure (mPAP) of 46 mmHg, pulmonary arterial wedge pressure (PAWP) of 12 mmHg, cardiac output 4.6 l/min, cardiac index 2.3 l/min/m^2^ and pulmonary vascular resistance of 591 dynes. Patient III:2 has improved under therapy to a mPAP of 55 mmHg, PAWP of 14 mmHg, cardiac output 6.0 l/min, cardiac index 2.9 l/min/m^2^ and pulmonary vascular resistance of 547 dynes.

**Table 1:** Clinical parameters

Parameter	II:4	III:2	III:4
Age at diagnosis	49	29	–
mPAP [mmHg] ^a^	55	63	–
PAWP [mmHg] ^b^	13	14	–
CO [l/min]	6.1	5.1	–
CI [l/min/m^2^]	3.2	2.4	–
PVR [dynes]	551	736	–
SaO_2_ [%]	97	98	96
sPAP [mmHg] at rest^ c^	94	47	20
sPAP [mmHg] during exercise ^c^	100	95	29
Peak VO_2_ [ml/min/kg]	18	17	24
RV area [cm^2^]	18	27	15
TAPSE [cm]	3.0	2.4	2.3
DLCO predicted [%]	77	60	91
NT-proBNP [ng/l]	89	58	31
6-MWD [m]	480	560	–
Medication	Silenafil, Macitentan	Sildenafil, Macitentan	–

^a^mPAP ≥25 mmHg characterises pulmonary hypertension

^b^PAWP >15 mmHg together with mPAP ≥25 mmHg characterises post-capillary PH due to left heart disease; PAWP ≤15 mmHg together with mPAP ≥25 mmHg characterizes pre-capillary PH

^c^sPAP >40 mmHg at rest and sPAP >45 mmHg at low workloads is considered here as abnormal and exercise induced pulmonary hypertension, respectively [27]. However, cut-offs are not clearly defined in current guidelines

*mPAP, (sPAP)* mean (systolic) pulmonary arterial pressure, *PAWP* pulmonary arterial wedge pressure, *CO* cardiac output, *CI* cardiac index, *SaO*_*2*_ oxygen saturation, *VO*_*2*_ oxygen uptake, *PVR* pulmonary vascular resistance, *RV* right ventricular, *TAPSE* tricuspid annular plane systolic excursion, *DLCO* diffusion capacity of the lung for carbon monoxide, *NT-proBNP* N-type pro brain natriuretic peptide, *6-MWD* 6 min walking distance

Pulmonary veno-occlusive disease (PVOD) was excluded in the patients on three grounds. Firstly, high resolution computer tomography showed no morphological changes typical for PVOD. Secondly, diffusion capacity of the lung for carbon monoxide (DLCO) values were greater than 50 % predicted. Values around 50 % are often seen in PVOD [26]. Thirdly, patients II:4 and III:2 have received PAH medication for 8 years and were stable under dual therapy. Whereas in PVOD a worsening of symptoms is often observed, once PAH medication has been given.

### Genetic findings

All living family members with manifest HPAH (II:4, III:2 in Fig. 2) carried a heterozygous mutations in *BMPR2* and *EIF2AK4*. In contrast, two non-diseased family members (III:1 and III:4) carried only the *BMPR2* mutation and no mutation in *EIF2AK4.* None of these family members developed manifest HPAH (Fig. 2) at of the time of this writing.

**Fig. 3:**
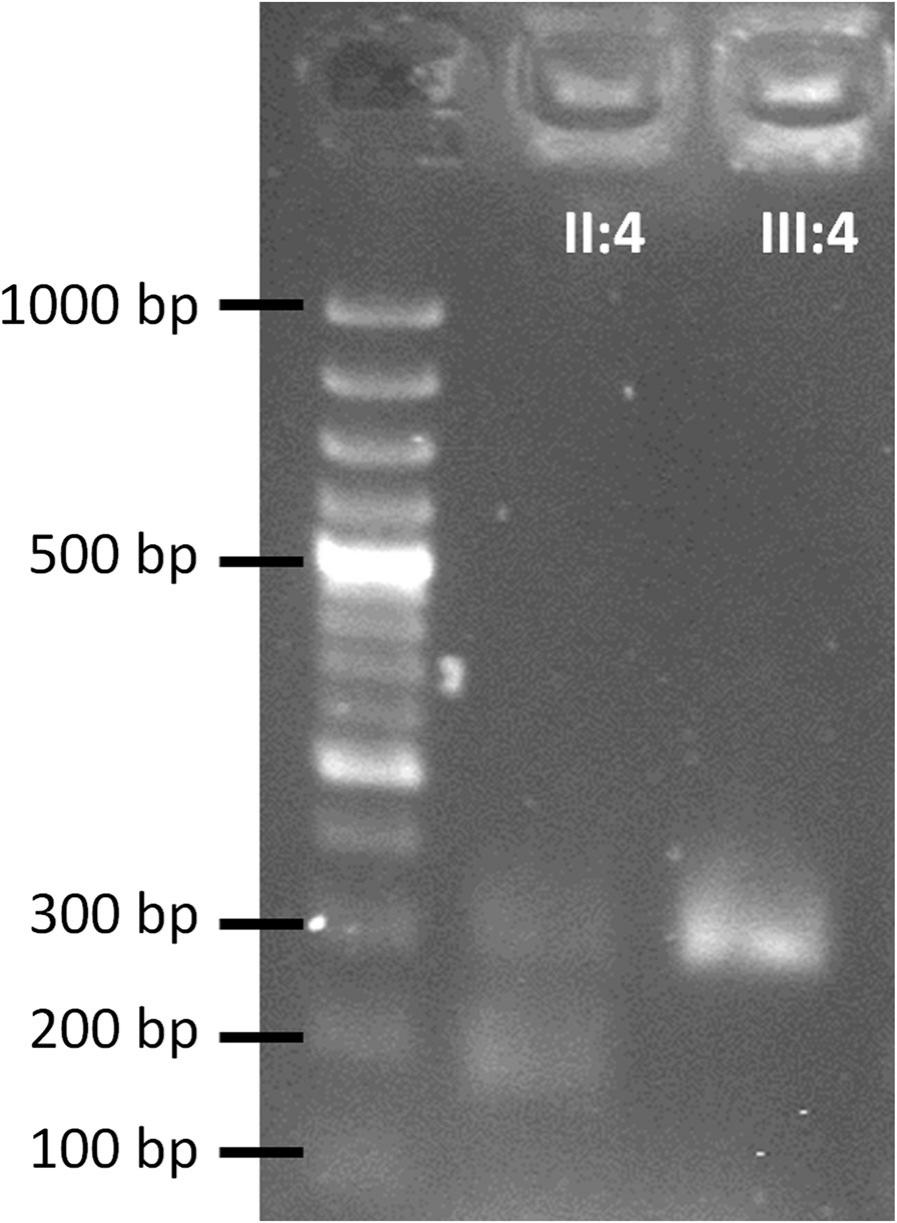
Effect of the *EIF2AK4* mutation c.4892+1G>T on cDNA level. Next to the ladder the affected individual *II:4* shows a heterozygous PCR product for the cDNA of *EIF2AK4* exons 36–39. The upper band shows the wildtype sequence (266 bp) while the lower band shows a product without exon 38 (147 bp). The healthy family member *III:4* is homozygous for the wild type PCR product indicating no loss of exon 38

The first familial mutation lay within exon 12 of the *BMPR2* gene (c.2308delC, p.(Arg770Glyfs*2)) and led to the deletion of a cytosine resulting presumably in a premature stop codon two amino acids downstream. The second mutation was located one base pair behind the end of exon 38 of the gene *EIF2AK4* (c.4892+1G>T) resulting in the loss of the complete exon 38 (Fig. 3). Due to this exon loss sequencing results suggested a frame shift and subsequent introduction of a premature stop codon in exon 39 at amino acid position 1599 instead of position 1650 in the regular protein; hence, a disrupted protein after the end of exon 37 is predicted.

While DNA was only available from five family members the pedigree analysis revealed two further obligate carriers of the *BMPR2* mutation. The grandfather (I:1) and the aunt (II:2) of the index patient must have been carriers for the cousin (III:1) to harbour the mutation. At the same time, the *EIF2AK4* mutation was most likely also present in the grandfather (I:1), who had died aged 49 years due to liver cirrhosis. Since the mother of the index patient (II:4) only developed manifest PAH at the age of 49 years, it is likely that the grandfather would have developed PAH due to the inferred presence of both mutations at a later stage in his life or had already developed the disease but had not been diagnosed until his death. Alternatively, the *EIF2AK4* mutation might have arisen *de novo* in II:4 or as a germ line mosaicism in either grandparent of the index patient.

In HPAH we expect a *BMPR2* mutation with a probability of 85 % [7, 8]. Under the null hypothesis stating that the mutation in *EIF2AK4* does not contribute to disease manifestation but instead represents a random event for the proband, the chance to observe the association of the two mutations has the probability of 0.00046. Taking the number of genes on the panel into account this association of both mutations under the null hypothesis reveals *p* = 0.00046*30 = 0.014, which is significant below the 5 % level. Hence, the co-occurrence of both mutations in affected family members is a significant association. Moreover, *EIF2AK4* co-segregates with disease conditioned on all *BMPR2* positive family members supporting the hypothesis of a second hit model, in which the diplotype of mutations in both genes has a high penetrance for PAH.

## Discussion

This is the first report of an autosomal dominantly inherited *EIF2AK4* mutation as second hit in a family with HPAH and known *BMPR2* mutation. Only those family members with a co-occurrence of a mutation in *BMPR2* and *EIF2AK4* were clinically affected and developed manifest HPAH, whereas carriers of the *BMPR2* mutation only had no symptoms of PAH. Thus, the results of this study offer an explanation for the reduced penetrance of the disease in this family and show that *EIF2AK4* may play a role also in families with autosomal dominantly inherited PAH.

### *EIF2AK4* mutation in autosomal dominant HPAH

*EIF2AK4* was first described in the autosomal recessively inherited pulmonary veno-occlusive disease (PVOD) [11] and pulmonary capillary haemangiomatosis [12]. Recently a single recessively inherited *EIF2AK4* mutation (c.3344C>T, p.(P115L)) was repeatedly identified in six consanguineous HPAH families with autosomal recessive mode of inheritance [14, 15]. Only homozygous mutation carriers developed the disease plus a single heterozygous carrier of a distinct *EIF2AK4* mutation, in whom the authors suspected a second non-identified mutation in the same gene [14]. Therefore, up to now *EIF2AK4* mutations have been believed to be a very rare in HPAH. Apart from the mentioned family we were able to identify a nonsense mutation in exon 8 in the *EIF2AK4* gene in another PAH patient with sporadic IPAH who had no other mutation in known candidate genes (data not shown). Thus, this gene might be more often affected than initially thought and contributes to the disease in an autosomal dominant manner. In contrast, *BMPR2* mutations occur in up to 85 % of familial cases and are autosomal dominantly inherited [7, 8]. However, many *BMPR2* gene carriers have no clinical symptoms and do not develop manifest PH even during a more than 10 year follow-up period [22]. Hence, the family described here provides an explanation for the decreased penetrance and suggests an autosomal dominantly contribution of *EIF2AK4* to disease manifestation.

### *EIF2AK4* mutation as “second hit” may explain variable penetrance

Up to date only four families with second hits have been described [19, 20]. This might be due to the fact that usually only 3 genes (*BMPR2, ACVRL1, ENG*) are analysed routinely in PAH patients in a sequential processes, i.e. if one mutation is discovered the other genes are not assessed. Thus, second hit mutations might be overlooked in general in the current routine diagnostic setting and particularly in genes such as *EIF2AK4*, which are usually not included in the diagnostic analysis.

While second hits are still rarely described in PAH this model is often found in other diseases such as the nephrotic syndrome [28] or the long QT-syndrome [29]. At the same time the decreased penetrance in PAH is an acknowledged pattern. Thus, second hits or modifier genes might be more common in PAH than known to date. We therefore contrast two genetic models: Firstly, we propose the “second hit model” to explain the low disease penetrance in PAH. In this model a single mutation in each gene on its own has a very low penetrance. Two mutations however, lead to a synergistic effect resulting in a high disease penetrance. The second model is the “single gene model”, representing the classical view for PAH suggesting *BMPR2* mutations alone are responsible for disease manifestation. Under this assumption, the *EIF2AK4* mutation would randomly occur in this family and not impact PAH manifestation. In the latter model the penetrance for the *BMPR2* variant must be moderate, since 4 (obligate) carriers of the mutation did not develop PAH up to ages 31, 37, 49 and 59.

Considering both models, it is significantly unlikely that both mutations in two known PAH genes occurred by chance in this family. Moreover, the *EIF2AK4* mutation clearly co-segregates with the disease in *BMPR2* positive family members suggesting a second hit model.

We have not observed an *EIF2AK4* mutation alone within this family, albeit in a different IPAH patient (data not shown) indicating at least a low penetrance to be present. Disease severity might moreover be influenced by the location of the respective mutations within the protein and thus their variable impact on protein function [8]. Therefore, we hypothesise according to the second hit model the penetrance to be intermediate if only *BMPR2* was positive, very low if only *EIF2AK4* was positive, and very high if *EIF2AK4* and *BMPR2* each harboured a mutation. However, a greater cohort study would be required assessing IPAH/HPAH patients for all known PAH genes to investigate the frequency of *EIF2AK4* mutations, second hits in PAH and their contribution to disease manifestation within affected families. Furthermore, animal studies would be required to investigate the proposed synergistic effect of two mutations in the same individual.

Not only the number of mutations in different genes but already the state (homo-/heterozygous) of the allele can affect disease severity [30] and even define which disease is developed. For example *BRCA2* may cause Fanconi aenemia in the homozygous state and familial breast and ovarian cancer in the heterozygous state [31]. In other diseases variants within the same gene might act dominantly or recessively depending on their localisation within the gene, e.g. *MAB21L2* can lead to eye malformations as a dominant or recessive trait [32]. Moreover, the 1000 genome project revealed around half a million variants in regulatory sites which most likely act as modifiers on gene expression and are not routinely considered in the diagnostic setting [33]. Thus, most Mendelian diseases are more complex than initially thought. The same most likely applies to PAH which is characterised by a reduced penetrance. Non-diseased mutation carriers may therefore only be provided with probabilities regarding disease manifestation by genetic counsellors. Any elucidation of further mechanisms refining the predictions of disease manifestation would reduce the uncertainty for patients and genetic counsellors. While we propose a synergistic effect of the two mutations, currently no different therapeutic approach is indicated. A close monitoring of these patients will be required to allow therapy escalation if necessary. A recent publication supports increased disease severity in patients with several mutations, younger age of onset and less effective treatment response in comparison to patients with a single mutation [34]. Thus, a comprehensive overview will be required with a large cohort of patients analysing current treatment options and genetic mutation status to re-evaluated current therapeutic strategies.

### Loss of protein function by *EIF2AK4* mutation

The gene *EIF2AK4* encodes a kinase termed general control nonderepressable 2 (GCN2) which phosphorylates the eukaryotic translation initiation factor 2α leading to a global down regulation of protein synthesis in response to amino acid starvation, hypoxia and viral infection but the up-regulation of specific stress response proteins [35]. Gene expression is increased in smooth muscle cells in the vessel wall and interstitial tissue [11]. While the gene function has been studied a clear link to PVOD or PAH still remains to be detected. However, an interaction between *EIF2AK4* and the BMPR2 pathway genes *SMAD1, SMAD4, ACVRL1* and *ENG* has been observed [11, 36]. Thus, an impaired functioning of both, BMPR2 and GCN2 (*EIF2AK4*), might potentiate its effect on the transcription of target genes of the BMPR2 pathway.

The *EIF2AK4* mutation of this family leads to the loss of a splice site and subsequently the loss of exon 38, presumably a frame shift and premature stop codon. In the last exons of the functional gene (31–39) lies the ribosomal binding domain and the dimerisation domain between amino acids 1396–1643 [37]. The last 51 amino acids of this domain were missing or partly exchanged by wrong amino acids in the affected members of this HPAH family. The domain is essential to recruit ribosomes for protein synthesis [38], thus a partial deletion will at least moderately affect protein-ribosome binding if not fully impair it. Moreover, in the same region the dimerisation domain is located. This facilitates the formation of a homodimer (2 EIF2AK4 proteins bind to each other) and thus a functional protein [38]. The formation of homodimers has been shown to be conserved in mice and yeast [39]. Single amino acid substitutions in the C-terminal domain in yeast already led to an inability of the protein to dimerise and to be functionally active [38]. The gene sequence of the C-terminal domain is highly conserved from mice to mammals suggesting corresponding functional impairments in humans [40]. A total deletion of this region in our HPAH family thus likely leads to a loss of function in the mutated gene.

## Conclusions

We were able to show *EIF2AK4* contributes to disease manifestation in this HPAH family in an autosomal dominant manner. We report a new mutation within *EIF2AK4* leading to HPAH as a second hit together with a mutation in *BMPR2* providing an explanation for the observed penetrance in this family. Only those family members with a co-occurrence of two mutations developed manifest HPAH. The occurrence of several mutations in PAH associated genes might be more frequent than initially thought. Thus, a simultaneous assessment of all PAH associated genes in more patients might shed light on the long standing question surrounding the reduced penetrance.

## Abbreviations

6-MWD: 6 min walking distance
BMPR2: Bone morphogenic protein receptor 2
cDNA: Copy DNA
CI: Cardiac index
CO: Cardiac output
DLCO: Diffusion capacity of the lung for carbon monoxide
EIF2AK4: Eukaryotic Translation Initiation Factor 2 Alpha Kinase 4
ExAC: Exome Aggregation Consortium
HPAH: Hereditary pulmonary arterial hypertension
IPAH: Idiopathic pulmonary arterial hypertension
mPAP: Mean pulmonary arterial pressure
NGS: Next generation sequencing
NT-proBNP: N-type pro brain natriuretic peptide
PAWP: Pulmonary arterial wedge pressure
PVOD: Pulmonary veno-occlusive disease
PVR: Pulmonary vascular resistance
RV: Right ventricular
SaO_2_: Oxygen saturation
sPAP: Systolic pulmonary arterial pressure
TAPSE: Tricuspid annular plane systolic excursion
VO_2_: Oxygen uptake
